# Underground railway particulate matter and susceptibility to pneumococcal infection

**DOI:** 10.1016/j.ebiom.2022.104063

**Published:** 2022-05-19

**Authors:** Lisa Miyashita, Rebecca Shears, Gary Foley, Sean Semple, Aras Kadioglu, Jonathan Grigg

**Affiliations:** a Centre for Genomics and Child Health, Blizard Institute, Queen Mary University of London, 4 Newark Street, London E1 2AT, UK; b The Department of Clinical Infection, Microbiology and Immunology, University of Liverpool, Liverpool, UK; c Institute for Social Marketing and Health, University of Stirling, Scotland

**Keywords:** London underground, Particulate matter, Pneumococcal infection

## Abstract

**Background:**

Concentrations of particulate matter less than 10 microns (PM_10_) on underground railways are higher than those near urban roads. Traffic-related PM_10_ increases pneumococcal infection via increasing the expression of platelet-activating factor receptor (PAFR), a receptor co-opted by pneumococci to adhere to cells. To date, it is unknown whether underground railway PM_10_ increases pneumococcal infection. This study sought to determine the effect of London Underground (LU) PM_10_ on; i) pneumococcal adhesion to airway cells, and ii) susceptibility to pneumococcal disease.

**Methods:**

A549 cells and human primary airway epithelial cells were cultured with 20 µg/mL PM_10_ from the Bakerloo (B-PM_10_) and Jubilee (J-PM_10_) line platforms of Baker Street station. PAFR expression was assessed by flow cytometry, and pneumococcal adhesion by colony forming unit (CFU) counts. Traffic-related PM_10_ was collected next to a main road near the station's entrance. The PAFR blocker CV3988 and the antioxidant N-acetyl cysteine were used to assess the role of PAFR-mediated pneumococcal adhesion and oxidative stress respectively. Pneumococcal infection of mice was done after exposure to 3×80 μg doses of intranasal LU-PM_10_.

**Findings:**

In A549 cells, human primary nasal cells, and human primary bronchial epithelial cells, B-PM_10_ and J-PM_10_ increased PAFR expression and pneumococcal adhesion. Stimulated adhesion was abrogated by CV3988 and N-acetyl cysteine. Traffic-related PM_10_ stimulated increased adhesion compared with B-PM_10_. B-PM_10_ and J-PM_10_ increased lung and blood CFU and mortality in mice. Treatment of B-PM_10_-exposed mice with CV3988 reduced blood CFU.

**Interpretation:**

LU-PM_10_ increases pneumococcal adhesion to airway cells and susceptibility to invasive disease in mice.

**Funding:**

The Medical College of Saint Bartholomew's Hospital Trust, and the UK Medical Research Council Programme Grant (MR/P011284/1).

**Research in context**

**Evidence before this study**

A literature review done in 2021 by the UK Committee on the Medical Effects of Air Pollution into the health effects associated with exposure to particulate matter (PM) in the London Underground concluded that; i) mass concentrations of PM at the platforms on London Underground lines are typically much higher than in ambient air, and ii) differences between underground PM and that found in ambient air mean that it is not possible to determine the nature and extent of any health risk to those travelling on the London Underground. Since traffic-related PM is associated with increased risk of pneumococcal pneumonia, and mechanistic studies report increased adhesion of *Streptococcus pneumoniae* (the pneumococcus) to airway cells exposed to PM *in vitro*, we searched PubMed with combinations of “bacterial infection”, “pneumonia”, “pneumococcus” and either “underground railway” or “subway”. No publications on the effects of PM on susceptibility were identified. A critical review of the health effects of PM air pollution in underground railway systems published in 2019 also reported no studies of susceptibility to bacterial infection.

**Added value of this study**

We report that inhalable particulate matter (PM_10_) from 2 deep platforms of the London Underground railway system, via induction of oxidative stress, increases the expression of platelet activating factor receptor (PAFR) by airway epithelial cells *in vitro*. This in turn increases PAFR-mediated pneumococcal adhesion and entry of bacteria in cells. In a mouse model, London Underground PM_10_ increases mortality and the translocation of pneumococcus into the systemic circulation.

**Implications of all the available evidence**

Since PM generated in an underground railway system has the capacity to increase susceptibility to pneumococcal infection, this cannot now be considered a nuisance dust. Concentrations of PM in underground railways should therefore be reduced, and epidemiological studies of workers should include pneumococcal infection.

Alt-text: Unlabelled box

## Introduction

Concentrations of inhalable particulate matter (PM either less than 10 or 2.5 microns in aerodynamic diameter; PM_10_, PM_2.5_) in underground railway systems with electric trains are frequently in excess of World Health Organization's limits,^1^ and raises concerns about its potential to cause adverse health effects. For example, Luglio and colleagues^2^ recently reported a mean real-time PM_2.5_ concentration in the Port Authority Trans-Hudson subway (New York, US) of 779 µg/m^3^, including 1,700 µg/m^3^ in one station. In Europe, Martins and colleagues^3^ reported concentrations of PM_2.5_ in platforms of the Barcelona (Spain) subway up to 91 µg/m^3^, and Smith and colleagues^4^ reported a mean PM_2.5_ in the London Underground (LU) of 88 µg/m^3^ compared with overground traffic-related PM_2.5_ of 22 µg/m^3^.

Particulate matter generated in underground railway systems differs in composition from traffic-related PM, since it is from mechanical wear of train components, including wheels, brake blocks, and non-rolling stock sources including rail wear and not from the combustion of fossil fuels.^5^ Smith and colleagues^4^ reported that LU-PM_2.5_ comprises of 47% iron oxide,14% other metallic and mineral oxides, 11% organic carbon, and 7% elemental carbon with 21% of the unidentified mass likely to be silicates. Similarly, Luglio and colleagues^2^ reported that iron and total carbon comprised 80% of US subway PM_2.5_ mass. Thus compared with traffic-derived inhalable PM, underground PM has a much greater contribution from iron, principally iron oxide.^5^

To date, no study has modelled the effect of PM from underground railway systems on clinically-relevant health outcomes. However, Seaton and colleagues^6^ reported that 8 h culture of the A549 cell line with 100 µg/mL LU-PM_2.5_ non-significantly increased interleukin-8 release, and concluded that LU-PM_2.5_ was of “no serious concern” to either workers or commuters. By contrast, a more recent review of studies of underground PM by Loxham and Nieuwenhuijsen^7^ concluded that underground PM has the capacity to elicit reactive oxygen species generation and induce inflammatory cytokine release from airway cells.

There is strong epidemiological evidence that exposure to traffic-related PM increases susceptibility to bacterial respiratory infection.^8^^,^^9^ Biological plausibility for this association is provided by our previous study which found that traffic-related PM_10_, via induction of oxidative stress, increases the expression of platelet-activating factor receptor (PAFR) on airway cells - the receptor co-opted by pneumococci to adhere to and infect cells.^10^ In the present study, we therefore sought to assess the effect of LU-PM_10_ on; (i) PAFR-dependent pneumococcal adhesion to human airway cells *in vitro*, and (ii) susceptibility to pneumococcal infection in a mouse model of asymptomatic nasal pneumococcal colonisation.^11^^,^^12^

## Methods

### Particulate matter

Collection of LU-PM_10_ was done using a high-volume cyclone^13^ placed on two of the deepest platforms of Bakerloo (B) and Jubilee (J) lines of Baker Street station, a major station at the junction of Baker Street and Marylebone Road (Supplementary Figure S1). Both lines are served by electric trains. Overground traffic-related PM_1__0_ was collected by placing the cyclone on the pavement of Marylebone Road within 50 m of Baker Street station's entrance. LU-PM_10_ was collected on different days, were pooled and stored at room temperature in a sterile glass container. Aliquots of PM_10_ were diluted in Dulbecco's phosphate-buffered saline (DPBS) to a final concentration of 1 mg/mL and stored as a master stock at -20^°^C.

### Airway cells

The human alveolar type II epithelial cell line A549 was purchased from Sigma-Aldrich (Poole, UK) and maintained in Dulbecco's Modified Eagle Medium (DMEM) supplemented with fetal bovine serum (FBS) and penicillin-streptomycin (Lonza, Basel, Switzerland). Passage number was less than 20. Human primary nasal epithelial cells (HPNEpC), and human primary bronchial epithelial cells (HPBEpC) and were purchased from PromoCell® (Heidelberg, Germany), and maintained in airway epithelial cell growth medium, with supplement kit, Primocin (InvivoGen, San Diego, USA), and 10% FBS. Passage number was less than 5. Cell membrane integrity was assessed by lactate dehydrogenase (LDH) release, according to the manufacturer's instructions (Sigma-Aldrich). Treatment of cells with distilled water was used as a positive control and indicated 100% LDH release.

### Platelet-activating factor receptor expression

Airway cells were seeded overnight into adherent cell culture plates (2×10^5^ cells per well) and cultured with LU-PM_10_ for 2 h before washing and detaching with trypsin. Cells were stained with an anti-PAFR primary antibody (1:200; ab104162 Abcam, Cambridge, UK, RRID:AB_10712285) for 1 h with shaking at room temperature. A PAFR isotype control (1:200; ab172730, Abcam, Cambridge, UK, RRID:AB_2687931) was included to control for nonspecific staining. The epithelial marker E-cadherin was included in all assays (1:100; ab1416, Abcam, Cambridge, UK, AB_300946). Cells were subsequently washed and stained with secondary antibodies conjugated to either Alexa Fluor 488 (1:3000; ab150077, Abcam, Cambridge, UK, AB_2630356) for detection of PAFR/isotype expression or allophycocyanin (1:1500; ab130786, Abcam, Cambridge, UK, RRID:AB_11160261) for detection of E-cadherin. Analysis was carried out on the BD FACS Canto II machine using BD FACSDiva software (BD Biosciences, Oxford, UK). PAFR is expressed as median fluorescence intensity (MFI).

### Pneumococcal adhesion

The *Streptococcus pneumoniae* type 2 encapsulated strain D39 (NCTC 7466) was purchased from the National Collection of Type Cultures (Central Public Health Laboratory, London, UK), grown to mid-logarithmic phase (OD_600_ = 0.4 to 0.6) in brain-heart infusion broth (BHI) (Oxoid, Basingstoke, UK) and stored at -80^°^C. Pneumococcal adhesion was conducted using an *in vitro* adhesion assay previously described.^10^ Briefly, airway epithelial cells were seeded overnight into adherent cell culture plates (2×10^5^ cells per well) and exposed to either LU-PM_10_ for 2 h, washed to remove PM, then *S. pneumoniae* D39 added at a multiplicity of infection (MOI) of 100 for a further 2 h to allow adhesion. Cells were subsequently washed to remove non-adherent bacteria, lysed, and plated for colony forming unit count (CFU/mL). In this assay, CFU count reflects both the number of pneumococci adherent to the surface of cells and the number of intracellular bacteria. To assess the intracellular fraction *per se*, adherent bacteria on the cell surface were first killed with penicillin (200 mg/mL) and gentamicin (10 mg/mL) for 30 min. Supernatant (10 µL) was plated on brain heart infusion agar to confirm that bacteria were killed. Intracellular pneumococci protected from antibiotic killing were recovered by cell lysis with ice-cold sterile water and plated on brain heart infusion agar to determine CFU.

The role of PAFR was determined by adding the PAFR receptor blocker CV3988 (half maximal inhibitory concentration [IC50] 0.28 μM^14^) to the pneumococcal adhesion assay at a final concentration of 20 μM, as previously reported.^15^ The role of PM-mediated oxidative stress was determined by adding N-acetyl cysteine (NAC; Sigma-Aldrich) at a final concentration of 5 mmol/L^12^ 30 min before and during exposure of cells to LU-PM_10_. N-acetyl cysteine was removed by washing cells prior to performing the pneumococcal adhesion assay.

### Murine infection model

Female CD1 mice (Charles River, Margate, UK) were maintained in individually ventilated cages at 22^°^C ± 1^°^C and 65% humidity with a 12 h light-dark cycle. Mice were acclimatised for 1 week before use and had free access to food and water. All procedures were carried out on age-matched mice aged 6 to 8 weeks or older. A total of 10 mice were included per group for all murine experiments as this is the required sample size to detect significant differences in CFU and survival. For all *in vivo* work, mice were randomly assigned to cages and cages were then randomly assigned to different treatment groups.

Mice were lightly anesthetised with a mixture of O_2_ and isoflourane and exposed once daily to either 80 μg LU-PM_10_ dissolved in 40 μL of PBS, or PBS alone, via the intranasal route for the duration of the experiment. After three consecutive daily LU-PM_10_ exposures, mice were mildly anaesthetised with 2.5% v/v Isofluorane over oxygen (1.4-1.6 L/min) before intranasal infection with 1×10^5^ CFU of D39 in 10 μL of PBS. Control mice were treated with 10 μL PBS. This infection protocol normally results in asymptomatic pneumococcal nasal carriage with no seeding of bacteria into lungs or translocation to blood.^12^ In experiments to assess bacterial loads in lung and blood, CFU counts were determined in groups of mice which were separately allocated for humane killing at either 1, 2, 4, or 7 days post infection. Bacterial CFU were determined as described previously.^16^ To assess PAFR expression, mouse nasal epithelial cells were stained with PE antimouse/human CD324 (E-Cadherin,RRID: AB_2563039) and APC-anti-PAFR antibodies (RRID: Ab_AB_2563039). Gating on single cells is shown with the gating strategy for PAFR^+^ and E-Cadherin^+^ cells (Supplementary Figure S2). To confirm the role of PAFR, the antagonist CV3988 or PBS was administered to mice via the intravenous route (5 mg/kg) 1 h prior to infection and at 24 h post infection. For histologic analysis, lungs of mice 24 h post exposure to 3×80 μg LU-PM_10_ alone or PBS alone were fixed overnight in neutral buffered formalin (10% formalin) followed by 95% ethanol, stained with haematoxylin and eosin and imaged using an Aperio CS2 Slide Scanner (Leica, Milton Keynes, UK). Apeiro Image Scope v12.3.2.8013 was used to analyse images. To assess nasal epithelial PAFR expression, the nasopharynx was removed from each mouse and homogenised tissue processed as described previously.^16^ Nasal cells were washed and resuspended in DPBS containing 10% FBS before staining with PE antimouse/human CD324 (E-Cadherin, 1:100 dilution) antibody (Biolegend, London, UK) and PAFR primary antibody (Abcam, 1:200 dilution). The primary antibody was conjugated to APC using a Lightning-Link® Allophycocyanin (APC) kit, as per the manufacturer's protocol (Innova Biosciences, Cambridge, UK). A PAFR isotype control, conjugated to APC using the same method as the PAFR primary antibody, was used to exclude any nonspecific staining. Analysis was carried out on a BD FACS Canto machine using BD FACSDiva software (BD Biosciences, Oxford, UK) (Supplementary Figure S2).

### Statistics

Data are summarised as median (IQR), and analysed by either Kruskal-Wallis test with Dunn's multiple comparisons test, or Mann Whitney test. Survival analysis was analysed by log-rank Mantel-Cox test. Data from *in vitro* studies are from at least 5 separate experiments. Analysis were performed using Prism 9 (GraphPad Software Inc., La Jolla, CA, USA), and *p*<0.05 considered statistically significant. To observe statistical difference in pneumococcal CFU density over time, a group size of 9 is required, while for survival studies in pneumococcal pneumonia the required sample size is 7, calculated using methodology described by Shah^17^ for sample size calculations for dichotomous variables (time to death) and continuous variables (pneumococcal CFU density). Calculations were based on two-sided Fisher's exact tests. Calculations were performed for comparison-wise power of 90% at a significance level alpha=5%. Standard deviations and expected differences were estimated from previous experiments investigating survival or CFU density in pneumococcal-infected mice

### Ethics

All animal experiments were performed at the University of Liverpool in accordance with the Animal Scientific Procedures Act 1986 and with the prior approval of the UK Home Office (PPL: P86De83DA) and the University of Liverpool ethics committee.

### Role of funders

The funders had no role in study design, data collection, data analyses, interpretation, or writing of report.

## Results

### A549 cells

**Figure 1:**
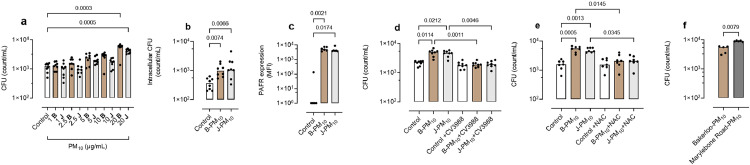
Effect of 20 µg/mL London Underground particulate matter less than 10 microns from microns from Bakerloo (B-PM_10_) and Jubilee lines (J-PM_10_) on A549 cells, (a) dose-response of London Underground particulate matter less than 10 microns in aerodynamic diameter from the Bakerloo (B-PM_10_) and Jubilee (J-PM_10_) lines on pneumococcal adhesion. Increased pneumococcal adhesion is reflected by increased colony forming unit (CFU) count, and is significant at 20 µg/mL, (B-PM_10_ and J-PM_10,_ n=8, technical replicates (TR)=3, =0.0003 and p=0.0005, respectively, (b) effect of 20 µg/mL PM_10_ on intracellular pneumococcal CFU count, assessed after treatment of cells with antibiotics to kill cell surface bacteria (B-PM_10_ and J-PM_10,_ n=8, TR=3, p=0.007 and 0.006 respectively), (c) effect of B-PM_10_ and J-PM_10_ on platelet-activating factor receptor (PAFR) expression (B-PM_10_ and J-PM_10,_ n=6, TR=2, p=0.002 and 0.017 respectively), (d) effect of the PAFR blocker CV3988 on pneumococcal adhesion (B-PM_10_ and J-PM_10,_ n=8, TR=3, p=0.001 and p=0.004 respectively), (e) effect of the antioxidant N-acetyl cysteine on pneumococcal adhesion, (B-PM_10_ and J-PM_10,_ n=7, TR=3, p=0.014 and 0.034, respectively), (f) comparison of adhesion stimulated by 20 µg/mL B-PM_10_ and 20 µg/mL overground Marylebone Road PM_10_, (n=5, TR=3, p=0.007) Expression was determined by flow cytometry and expressed as median fluorescence intensity (MFI). Columns represent medians and p values are calculated by either Kruskal-Wallis with *post hoc* multiple comparison testing, or by Mann Whitney test.

In A549 cells, B-PM_10_ and J-PM_10_ (LU-PM_10_) increased pneumococcal adhesion at 20 µg/mL (Figure 1a, p=0.0003 and p=0.0005 respectively Kruskal-Wallis with *post hoc* multiple comparison testing). LU-PM_10_ at 20 µg/mL did not increase LDH release (data not shown), and this concentration was used in subsequent airway cell experiments. B-PM_10_ and J-PM_10_ increased intracellular pneumococci, assessed after killing adherent cell-surface bacteria (Figure 1b, p=0.007 and 0.006 respectively Kruskal-Wallis with *post hoc* multiple comparison testing). Exposure of A549 cells to B-PM_10_ and J-PM_10_ increased PAFR expression (Figure 1c, p=0.002 and p=0.017 respectively, Kruskal-Wallis with *post hoc* multiple comparison testing*)* and the PAFR blocker CV3988 abrogated both B-PM_10_ and J-PM_10_-stimulated pneumococcal adhesion (Figure 1d, p=0.001 and p=0.004 respectively, Kruskal-Wallis with *post hoc* multiple comparison testing).

In A549 cells, the antioxidant NAC abrogated B-PM_10_ and J-PM_10_-stimulated adhesion (Figure 1e, p=0.014 and 0.034 respectively Kruskal-Wallis with *post hoc* multiple comparison testing). CV3988 and NAC had no effect on pneumococcal adhesion to unstimulated cells (Figure 1d,e Kruskal-Wallis with *post hoc* multiple comparison testing). Pneumococcal CFU counts stimulated by overground traffic-related PM_10_ were higher compared with CFU after incubation with the same concentration of B-PM_10_ (Figure 1f, p=0.007 Mann Whitney test).

### Human primary airway cells

**Figure 2:**
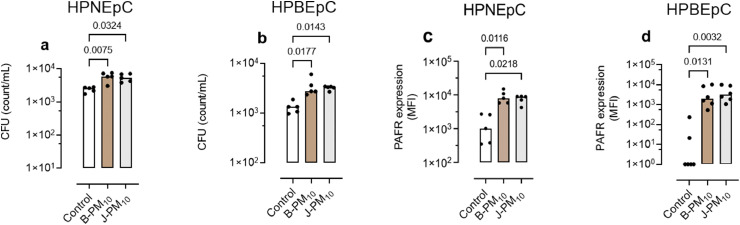
Effect of 20 µg/mL London Underground particulate matter less than 10 microns from microns from Bakerloo (B-PM_10_) and Jubilee lines (J-PM_10_) on human primary epithelial cells. Pneumococcal adhesion to (a) human primary nasal epithelial cells (HPNEpC B-PM_10_ and J-PM_10_ n=5, technical replicates (TR)=3, p=0.007 and p=0.032, respectively, and (b) primary bronchial epithelial cells (HPBEpC; B-PM_10_ and J-PM_10_ n=5, TR=3, p=0.017 and p=0.014, respectively). Platelet-activating factor receptor (PAFR) median fluorescence intensity (MFI) expression, (c) HPNEpC (B-PM_10_ and J-PM_10_, n=5, TR=2, p= 0.011 and p=0.02, respectively) and, (d) HPBEpC (B-PM_10_ and J-PM_10_, n=5, TR=2, p=0.013 and p=0.003, respectively. Columns represents median and p values are calculated by Kruskal-Wallis with *post hoc* multiple comparison testing.

B-PM_10_ and J-PM_10_ increased pneumococcal adhesion to human primary nasal epithelial cells (HPNEpC, Figure 2a, p=0.007 and p=0.032 respectively), and to human primary bronchial epithelial cells (HPBEpC, Figure 2b, p=0.017 and p=0.014 respectively Kruskal-Wallis with *post hoc* multiple comparison testing). B-PM_10_ and J-PM_10_ increased PAFR expression by both HPNEpC (Figure 2c, p=0.02 and p=0.011 respectively Kruskal-Wallis with *post hoc* multiple comparison testing), and HPBEpC (Figure 2d, p=0.013 and p=0.003 respectively).

### Murine infection model

**Figure 3:**
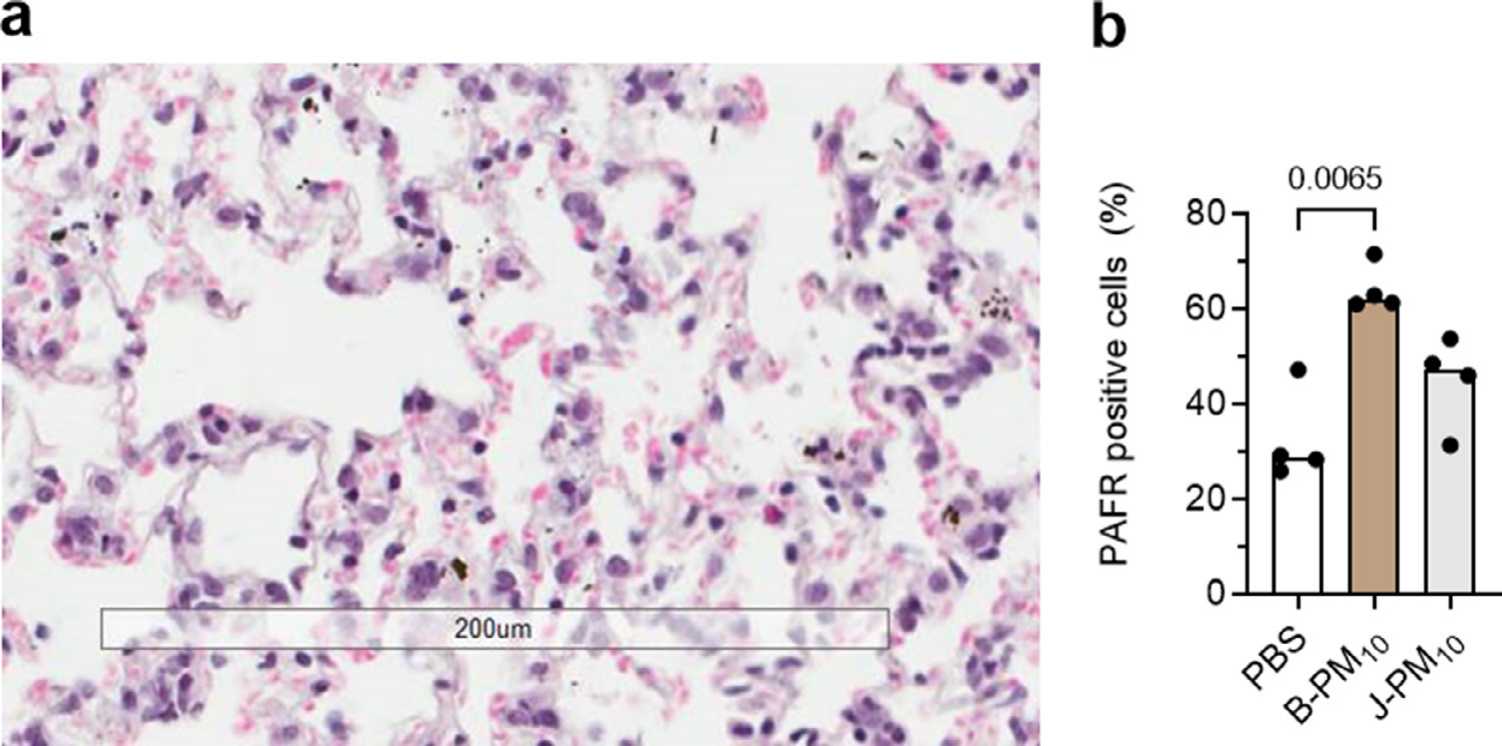
Effect of intranasal instillation of three doses of 80 µg London Underground railway (LU) particulate matter less than 10 microns from Bakerloo (B-PM_10_) and Jubilee lines (J-PM_10_) in female CD1 mice. (a) Photomicrograph shows a representative section of a mouse lung 24 h post instillation. There are free particles (black spots) in alveoli, and intracellular particles. Bar represents 200 μm, (b) platelet-activating factor receptor (PAFR) positive nasopharyngeal cells (n=4 per group, B-PM_10_ and J-PM_10,_ p=0.006 and p>0.05, respectively). Columns represents median and p values are calculated by Mann Whitney test.

**Figure 4:**
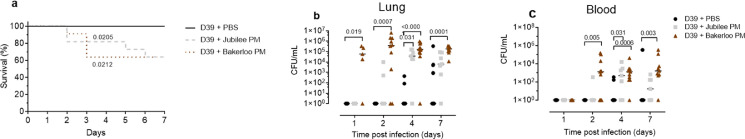
Effect of London Underground particulate matter less than 10 microns in aerodynamic diameter from Bakerloo (B-PM_10_) and Jubilee lines (J-PM_10_) on pneumococcal infection in female CD1 mice. Mice were exposed to either intranasal PBS, or 3 x intranasal doses of 80 μg B-PM_10_ or J-PM_10_. Mice were then infected with 10^5^ CFU of D39 *S. pneumoniae*; (a) probability of survival of animals (n=10 mice per group, p=0.021 and p=0.020, respectively), (b) lung colony forming unit (CFU) counts(p=0.031 and p<0.0001) respectively, n=5 per group allocated to day 1, and n=10 to n=15 per group allocated to days 2, 4, and 7, (c) blood colony forming unit (CFU) counts (p=0.031 and p=0.0006 respectively), n=5 per group allocated to Day 1, and n=10 to n=15 per group allocated to days 2, 4, and 7. Columns indicates median and p values calculated by Kruskal-Wallis with *post hoc* multiple comparison testing between groups for each day.

Intranasal instillation of LU-PM_10_ to mice deposited PM in the lower airway (Figure 3a). Uninfected mice exposed to LU-PM_10_ remained asymptomatic for up to 7 days (data not shown, n=10 mice per group). Consistent with our established model^11^^,^^12^, all mice (n=10 per group) treated with PBS, and then infected with pneumococci, remained asymptomatic up to 7 days (Figure 4a). By contrast, mortality was observed in B-PM_10_ and J-PM_10_-exposed mice infected with pneumococci (Figure 4a, p=0.021 and p=0.020 respectively, n=10 per group Kruskal-Wallis with *post hoc* multiple comparison testing).

Consistent with increased mortality with exposure to LU-PM_10_, both lung and blood pneumococcal CFU counts were increased after B-PM_10_ and J-PM_10_ (Figure 4 b, c). Although B-PM_10_-exposed mice exhibited a more rapid increase in lung and blood CFU counts (Figure 4 b,c), by day 4 both B-PM_10_ and J-PM_10_ exposure increased lung CFU (p=0.031 and p<0.0001 respectively Kruskal-Wallis with *post hoc* multiple comparison testing) and blood CFU (Figure 4 b,c, p=0.031 and p=0.0006 respectively Kruskal-Wallis with *post hoc* multiple comparison testing).

To assess the role of PAFR, nasal epithelial PAFR expression was assessed in uninfected mice (n=4 per group) 24 h after 3 intranasal doses of LU-PM_10_ (i.e. at the infection timepoint). Instillation of B-PM_10_ increased nasal epithelial PAFR expression (Figure 3b, p=0.006 Mann Whitney test), but there was no significant effect J-PM_10_ on nasal PAFR (Figure 3b, p>0.05 Mann Whitney test).

**Figure 5:**
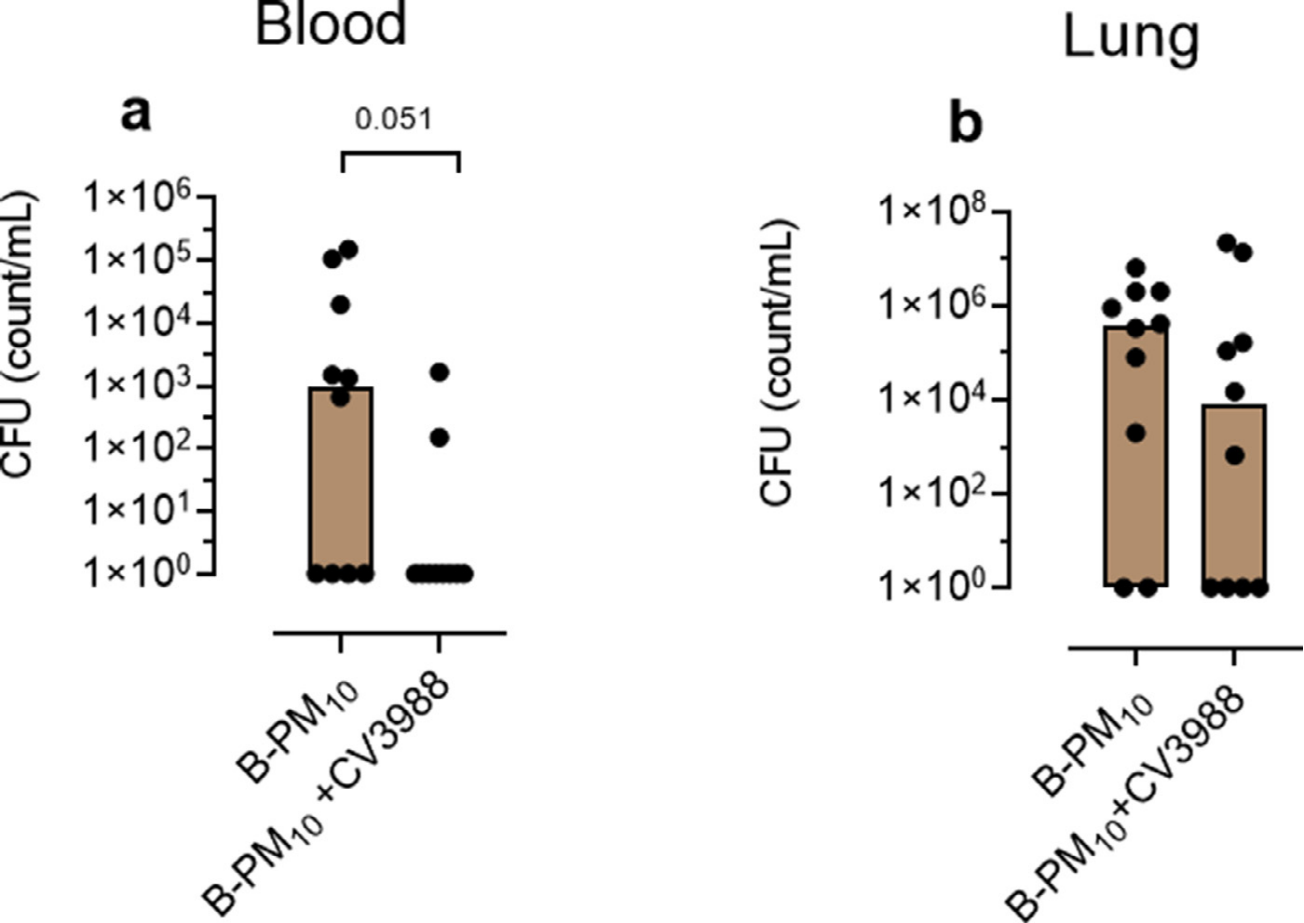
Effect of intravenous treatment of mice with 5 mg/kg of the PAFR blocker CV3988. Female CD1 mice (n=10 per group) were exposed to intranasal 3×80 μg Bakerloo (B)-PM_10_. The PAFR antagonist CV-3988 or PBS (control) was then administered intravenously 1 h prior to infection with 10^5^ colony forming units (CFU) of D39 *S pneumoniae* infection, and 24 h post infection. Animals were sacrificed at 2 days post infection; (a) blood CFU counts (p*=*0.051), (b) lung CFU counts (p>0.05). Columns indicates medians and p values are calculated by Mann Whitney test.

To assess the role of PAFR, we performed a further pneumococcal infection experiment using B-PM_10_ exposure alone. Intravenous treatment of B-PM_10_-exposed mice with the PAFR blocker CV3988 (n=10 per group) reduced subsequent blood CFU counts (Figure 5a, p*=*0.051 Mann Whitney test), but not lung CFU counts (Figure 5b, p>0.05 Mann Whitney test).

## Discussion

In this study we found that PM_10_ sampled from platforms of two deep lines of the London Underground, increased pneumococcal adhesion and penetration into airway cells *in vitro*. Increased adhesion was PAFR-dependent since LU-PM_10_ increased PAFR expression, a receptor the bacterium co-opts to adhere to cells and move into the cytoplasm as the receptor is internalised,^18^ and increased adhesion was abrogated by the PAFR blocker CV3988. Consistent with previous reports that PAFR does not mediate low levels of pneumococcal adhesion to unstimulated airway cells *in vitro*,^18^ CV3988 had no effect on adhesion to control cells.

The initial stimulus for increased PAFR expression, as previously reported for traffic-derived PM,^10^ is oxidative stress, since adding the antioxidant N-acetyl cysteine with LU-PM_10_ completely attenuated increased adhesion. Furthermore, these results are compatible with Karlsson and colleagues,^19^ who reported that PM from the Stokholm subway increases intracellular reactive oxygen species in airway cells. LU-PM_10_ also increased adhesion and PAFR expression to human primary nasal and bronchial airway cells, confirming its capacity to increase pneumococcal adhesion to both upper and lower respiratory tract epithelial cells i.e. the initial sites of infection.

To model the complexity of host responses to *S. pneumoniae in vivo*, we assessed the effect of LU-PM_10_ in a murine pneumococcal infection model which normally results in asymptomatic nasal colonisation.^12^ As expected, mice in the present study infected with pneumococci alone remained asymptomatic. We also established that intranasal instillation of LU-PM_10_ exposed not only in the upper but also the lower airway, and that LU-PM_10-_exposed, uninfected, mice remained asymptomatic. A remarkable finding in this non-lethal nasopharyngeal carriage model,^12^ was that exposure of mice to LU-PM_10_ increased both mortality from pneumococcal infection, and increased translocation of bacteria into the systemic circulation. A role for PAFR in mediating pneumococcal translocation in this model is suggested by; i) reduced blood CFU after PAFR blocker therapy (CV3988), and ii) the capacity of B-PM_10_ to increase nasal epithelial PAFR expression. However, the site of PAFR-dependent translocation of bacteria into the systemic circulation remains unclear since we did not assess sequential changes in PAFR and CFU throughout the whole respiratory tract pre- and post-infection. In addition, that J-PM_10_ did not increase nasal PAFR but did increased mortality from pneumococcal infection, suggests that susceptibility to infection in this model is not only due to upregulation of PAFR.

There are limitations to this study. First, although the PM concentration used in mice was similar to that of previous studies into PM-mediated susceptibility to infection,^20^^,^^21^ whether three intranasal doses of 80 μg LU-PM_10_ reflects human exposure during regular commuting or occupational exposure, is unclear. However, we speculate that the risk of developing pneumococcal infection is increased after repeated exposure to 800 μg/m^3^ inhalable PM reported in some LU platforms.^4^ Second, few epidemiological studies have focussed on the health effects of underground railway PM. Assuming a similar toxicity to outdoor PM_2.5_, Luglio and colleagues^2^ estimated that subway commuters exposed to subway PM_2.5_ have 11% increased risk of cardiovascular death. By contrast, a recent systemic review of studies of underground railway workers, concluded that there was little or no association of either disease or biomarker endpoints, albeit small sample sizes meant that it was difficult to draw robust conclusions.^7^ Our finding that traffic-related PM_10_ simulated higher levels of pneumococcal adhesion to airway cells compared with the same concentration of LU-PM_10_, suggests that direct extrapolation from epidemiological studies into the health effects of outdoor PM is difficult. Third, the sample of LU-PM_10_ contained a mixture of particle fractions less than 10 microns in aerodynamic diameter (e.g. PM_2.5_ and ultrafine), and we have not determined the effect of these smaller sized fractions on susceptibility to infection. Fourth, all assays were conducted using one *S. pneumoniae* strain (D39), however, PAFR-dependent adhesion to cells has been described for a wide range of pneumococci including R6, clinical isolate strain 132, TIGR4 and 19F.^18^^,^^22^^,^^23^ Furthermore, we previously reported comparable levels of infection when using D39 and the highly invasive serotype 1 strain in both *in vitro* and *in vivo* experiments to investigate the effect of airborne dust on invasive pneumococcal disease.^24^

In conclusion, our results suggest that epidemiological studies of workers and commuters frequently using underground railway systems should include susceptibility to pneumococcal infection, and that nasal PAFR expression has potential as an exposure biomarker in this regard.

## Funding

The Medical College of Saint Bartholomew's Hospital Trust, and the UK Medical Research Council Programme Grant (MR/P011284/1).

## Contributors

Study conception and design-LM, RS, GF, SS, AK, JG; data acquisition-LM, RS; data analysis-LM, RS, AK, JG.; data interpretation LM, RS, GF, SS, AK, JG.; data verification-LM, RS, AK, LG.; manuscript drafting and revising-LM, RS, GF, SS, AK, JG. JG and AK vouch for these data. All authors have read and approved the final version of this manuscript.

## Data sharing statement

All data collected for the study and a data dictionary defining each field in the set will be made available without restriction. These data will be available with publication. Data as original Graph Pad prism files will be made available by emailing the corresponding author (j.grigg@qmul.ac.uk).

## Declaration of interests

JG reports personal payment to provide expert medical evidence at a UK inquest related to air pollution, and is a member of the UK Committee on the Medical Effects of Air Pollution. There are no disclosures for the other authors.

## Appendix Supplementary materials

Image, application 1
