# Supplementary material for: Underground railway particulate matter and susceptibility to pneumococcal infection
Source: eBioMedicine. 2022 May 19;80:104063. doi: 10.1016/j.ebiom.2022.104063 (PMC9127572; doi:10.1016/j.ebiom.2022.104063)
Supplement: Supplementary file 1 [file mmc1.docx]

**
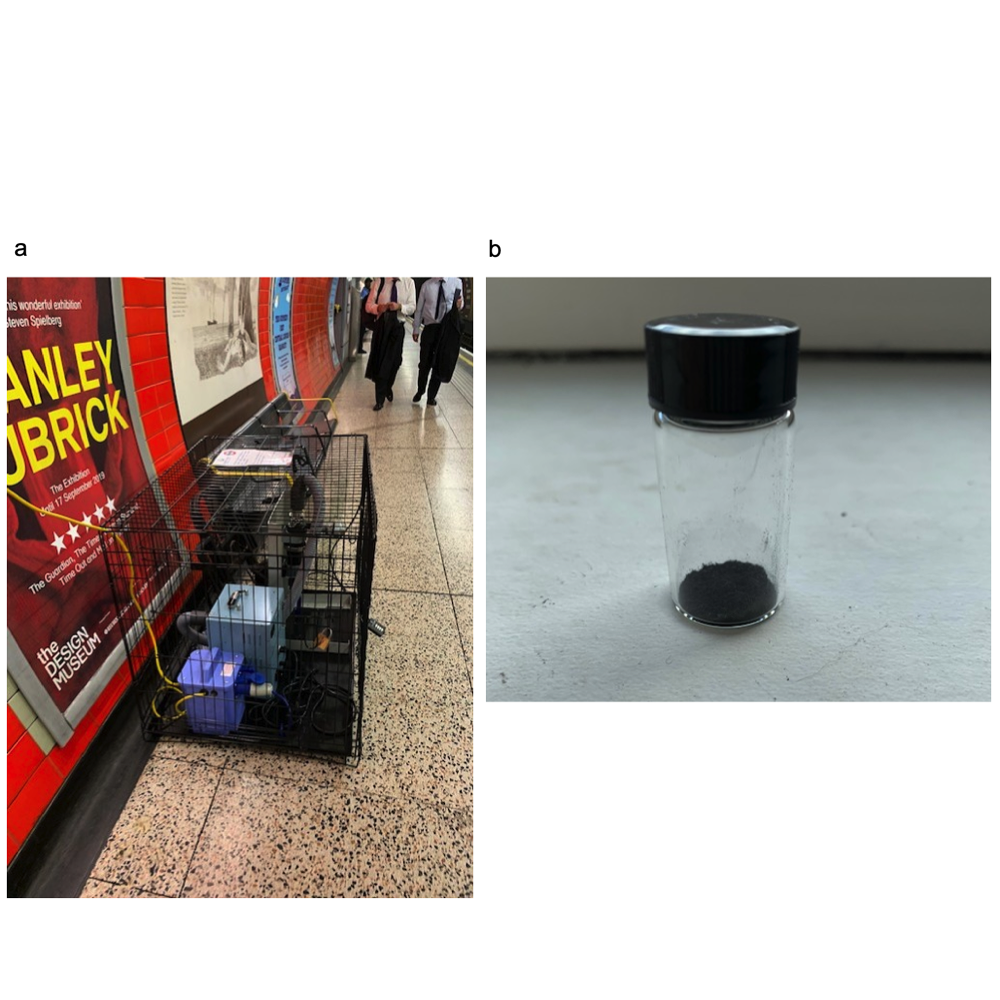
**

**Supplementary Figure S1.** a) London Underground particulate matter less than 10 microns (LU-PM_10_) collection by a cyclone situated on a platform of Baker Street station, b) Resulting black LU-PM_10_ collected by the cyclone.

**
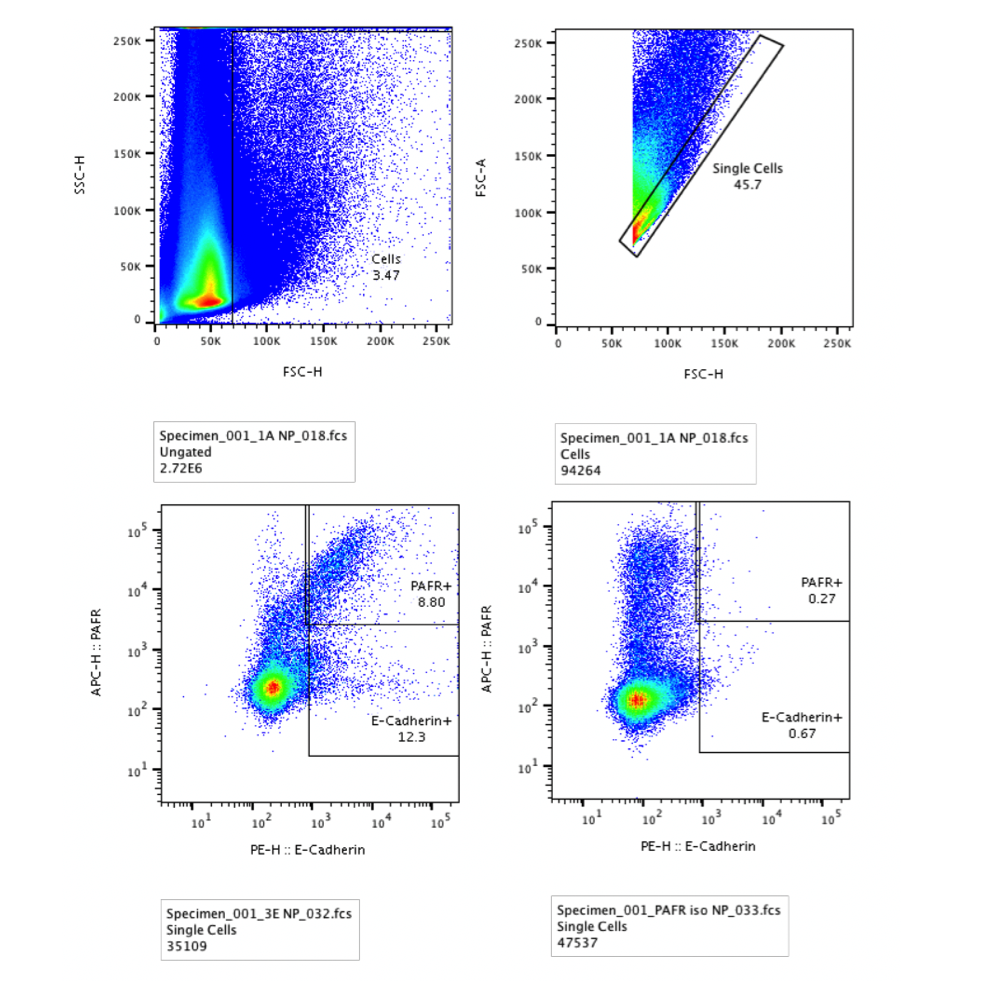
**

**Supplementary Figure S2.** Gating strategy for platelet activating factor receptor (PAFR) positive epithelial cells in the mouse nasopharynx. To assess PAFR expression, mouse nasal epithelial cells were stained with PE antimouse/human CD324 (E-Cadherin) and APC-anti-PAFR antibodies. Gating on single cells is shown (top right) and gating strategy for PAFR^+^ and E-Cadherin^+^ cells (bottom left). PAFR isotype control is shown in bottom right.
